# Comparative Analysis of Genome Wide DNA Methylation Profiles for the Genic Male Sterile Cabbage Line 01-20S and Its Maintainer Line

**DOI:** 10.3390/genes8060159

**Published:** 2017-06-16

**Authors:** Fengqing Han, Xiaoli Zhang, Xing Liu, Henan Su, Congcong Kong, Zhiyuan Fang, Limei Yang, Mu Zhuang, Yangyong Zhang, Yumei Liu, Zhansheng Li, Honghao Lv

**Affiliations:** 1 Institute of Vegetables and Flowers, Chinese Academy of Agricultural Sciences, Key Laboratory of Biology and Genetic Improvement of Horticultural Crops, Ministry of Agriculture, 12# Zhong Guan Cun Nandajie Street, Beijing 100081, China; feng857142@163.com (F.H.); zxl19871009@163.com (X.Z.); 13161176649@163.com (X.L.); 18810835083@163.com (H.S.); 13121238399@163.com (C.K.); fangzhiyuan@caas.cn (Z.F.); yanglimeicaas@163.com (L.Y.); zhuangmu@caas.cn (M.Z.); zhangyangyong@caas.cn (Y.Z.); liuyumei@caas.cn (Y.L.); lizhansheng@caas.cn (Z.L.); 2 Germplasm Innovation in Northwest China, Ministry of Agriculture, College of Horticulture, Northwest A&F University, Yangling 712100, China

**Keywords:** *Brassica oleracea*, DNA methylation, genic male sterility

## Abstract

Methylation modifications play an important role in multiple biological processes. Several studies have reported altered methylation patterns in male sterile plants such as rice and wheat, but little is known about the global methylation profiles and their possible roles in the cabbage (*Brassica* *oleracea*) male sterile line. In this study, single-base-resolution bisulfite sequencing (BS-Seq) was adopted to identify the pattern and degree of cytosine methylation in the male sterile line 01-20S and its near-isogenic fertile line 01-20F. Similar methylation patterns were profiled, with some changes observed in local positions. In total, 505 differentially methylated genomic regions (DMRs) and 106 DMR-associated genes were detected. Nine genes related to pollen development were discovered and further validated by a quantitative reverse-transcription polymerase chain reaction (qRT-PCR). Among these, four were downregulated in 01-20S. In particular, *Bol039180* (an invertase/pectin methylesterase inhibitor family protein) is likely involved in pectin degradation, and might play an important role in the pollen separation defects of 01-20S. This study facilitates a better understanding of DNA methylation alterations and their possible roles in genic male sterility in cabbages.

## 1. Introduction

DNA methylation is an epigenetic modification that plays important roles in chromatin conformation, transposon silence, and gene expression [1,2]. Cytosine methylation occurs in three sequence contexts of the cytosine residues: CG, CHG, and CHH, where H represents A, T, or C [3,4]. Methylation modifications are stable and heritable but may change in response to developmental and environmental cues [5].

Several pathways and key enzymes responsible for the maintenance and removal of DNA methylation have been reported in *Arabidopsis*. The maintenance of methylation suggests a possible dependence on nucleotide variations [6,7]. Methyltransferase 1 (MET1) is responsible for introducing methyl groups from the donor S-adenosine methionine specifically into the CG sequence context [8], whereas chromomethylase 3 (CMT3) is responsible for CHG methylation [9,10]. CHH de novo methylation is attributed to domain-rearranged MET1 and 2 (DRM1 and DRM2) [11,12]. This process is guided by 24-nt small interfering RNAs (siRNAs) and CMT2, depending on the interaction with decrease in DNA methylation 1 (DDM1) [11,12]. In reverse, the demethylation process is catalyzed by a series of enzymes, including the DNA demethylases DEMETER (DME), repressor of silencing 1 (ROS1), and DME-like 2 and 3 [13,14].

Clear evidence suggests that DNA methylation regulates many biological processes, including transposon silencing, genomic imprinting, DNA repair, and gene transcription [1,15]. Transposable elements (TEs) typically have high methylation levels, which are associated with inactive TEs and the transcription of neighboring genes [16]. CG methylation at the promoter region usually results in decreased gene expression [4]. Gene body methylation appears to be conserved across numerous species, and its role remains largely unclear [17].

Several studies have reported the status of DNA methylation during male gamete development [18,19,20]. A reprogramming of the DNA methylation occurs during pollen development, and the activity of TEs is regulated in gametes. Calarco et al. found that the plant germline retains CG and CHG DNA methylation but that CHH methylation is lost from retrotransposons and reprogrammed with guidance from 24-nt siRNAs [18]. This process is believed to contribute to epigenetic inheritance, transposon silencing, and imprinting [18,19,20]. Moreover, abnormal DNA methylation reprogramming in pollen may be associated with male gametophyte fertility [20,21,22]. In *Arabidopsis*, 5-methylcytosine DNA glycosylase DME is required for maternal allele demethylation and gene imprinting in the endosperm [20]. Notably, the DME-mutant showed abnormally high levels of CG methylation and defects in pollen viability, suggesting that DME-mediated demethylation plays a role in male fertility [21]. A transcriptome analysis of the maize male sterile mutation *ms8* revealed increased expression levels of methyl-binding domain protein before and during meiotic prophase I, which may be related to the incipient dissolution of the pollen mother cell (PMC) [22].

Furthermore, some studies have found evidence that DNA methylation regulates male sterility. In environmentally sensitive genic male sterile (EGMS) rice, Ding et al. [23] reported a long noncoding RNA named long-day-specific male-fertility-associated RNA (LDMAR), which is responsible for male sterility. Increased methylation was observed in the LDMAR promoter of Nongken 58S, and resulted in the reduced expression of LDMAR and male sterility. Thus, RNA-mediated DNA methylation may be involved in the regulation of male sterility. Recently, Chen et al. used the methylation-sensitive amplified polymorphism (MSAP) method to assess DNA methylation changes in the photoperiod- and thermo-sensitive male sterile rice strain PA64S [24]. A subsequent study revealed genome wide DNA methylation variation in another strain of photoperiod- and thermo-sensitive male sterile rice, Peiai 64S, using methylated DNA immunoprecipitation sequencing (MeDIP-seq) [25]. Both studies revealed some clues that DNA methylation regulates male sterility.

Some previous studies have reported the relationship between cytoplasmic male sterility (CMS) and DNA methylation status in rice [26] and maize [27]. Chen et al. observed higher DNA methylation levels in maize fertility-restored hybrids than in maize sterility-maintained hybrids and detected a specific site (16–1) in one of the restorer genes, *Rf5* [27]. This finding suggests that DNA methylation may regulate the expression of fertility restorer genes. DNA methylation and gene expression alterations in genic male sterile (GMS) mutations have also been studied in tomato [28] and wheat [29].

In the previous study we, for the first time, identified a sterile male cabbage mutant 79-399-3 that was the result of a single dominant gene named *Ms-cd1* [30]. *Ms-cd1* is slightly sensitive to temperature and is also influenced by different genomic backgrounds. Under permissive temperatures (i.e., relatively low temperatures), anthers of a few *Ms-cd1* background individuals can produce a few viable pollen grains. *Ms-cd1* was introduced to several elite lines to generate a male sterility line for a specific two-step hybrid cabbage breeding program. This breeding program has been successfully implemented in commercial hybrid cabbage seed production in China.

Some work has been performed to reveal the mechanism of this dominant male sterility (DGMS). Cytological observations of this male sterile type confirmed the presence of defects in the degradation of the PMC and the callose walls [31]. Several genes that were differently expressed during the pollen development stage were identified [31,32]. Furthermore, *Ms-cd1* was recently fine-mapped to a 39.4 kb region on C09 [33]. However, its molecular mechanism remains unclear.

In this study, high-throughput single-base-resolution bisulfite sequencing (BS-Seq) was adopted to identify the pattern and degree of cytosine methylation in the buds of the male sterile line 01-20S and its corresponding fertile line 01-20F. We aimed to detect methylation variations caused by the *Ms-cd1* background and to assess the possible roles of different methylation genes in male fertility.

## 2. Results

### 2.1. Methylation Landscape of F and S

Two libraries—F (01-20F) and S (01-20S)—were prepared. Single-base DNA methylation BS-Seq of the two libraries generated a total of 160,642,248 (F) and 146,248,884 (S) raw reads (125 bp). Following filtering, 48,951,106 clean reads (48.8%) in sample F and 43,435,200 clean reads (48.7%) in sample S were successfully and uniquely aligned to the ‘02-12’ cabbage reference genome (Table S1). These data were used to retrieve the methylation level for each CG, CHG, and CHH site.

**Figure 1:**
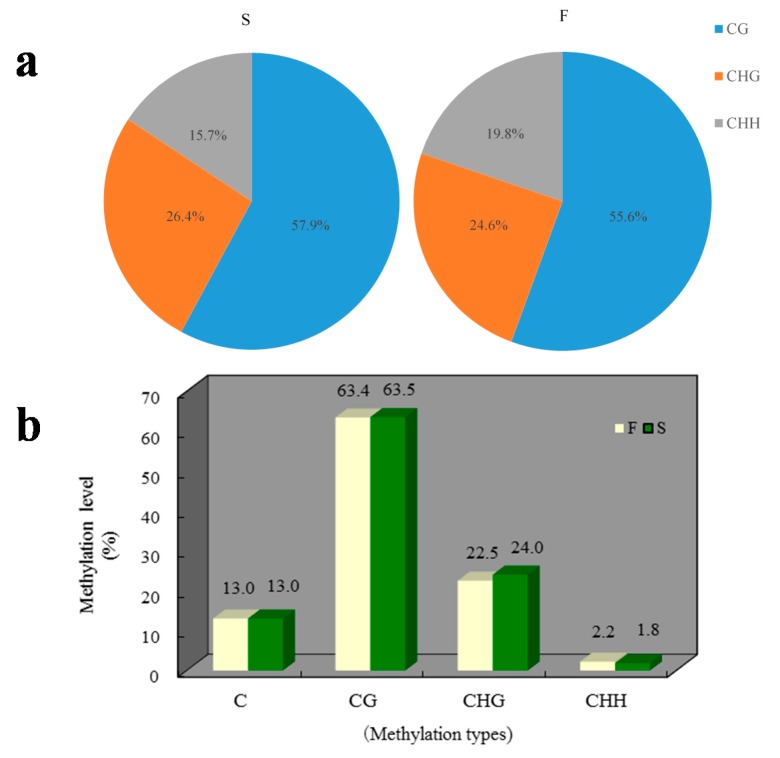
DNA methylation patterns of 01-20F (F) and 01-20S (S). (**a**) The cytosine methylation level (mC) percentage for each sequence context; (**b**) Levels of CG, CHG and CHH methylation in the whole cabbage genome.

A total of 7,059,969 cytosine methylation levels (mCs) (55.6% at CG sites, 24.6% at CHG sites and 19.8% at CHH sites, where H = A, T, or C) and 7,004,427 mCs (57.9% at CG sites, 26.4% at CHG sites and 15.7% at CHH sites) were identified in the F and S cabbage samples, respectively (Figure 1a). The total mCs were identical between the F (13.0%) and S (13.0%) samples (Figure 1b), whereas the DNA methylation levels in the three contexts differed. The overall genomic methylation degree of the mCHGs was significantly higher in the S samples (24.0%) than in the F samples (22.5%) (*p*-value < 0.01). For the mCHHs, the methylation level was significantly lower for S (1.8%) than for F (2.2%) (*p*-value < 0.01), whereas for the mCGs, the methylation level showed almost no differences between the F (63.4%) and S (63.5%) samples (*p*-value = 0.82).

**Figure 2:**
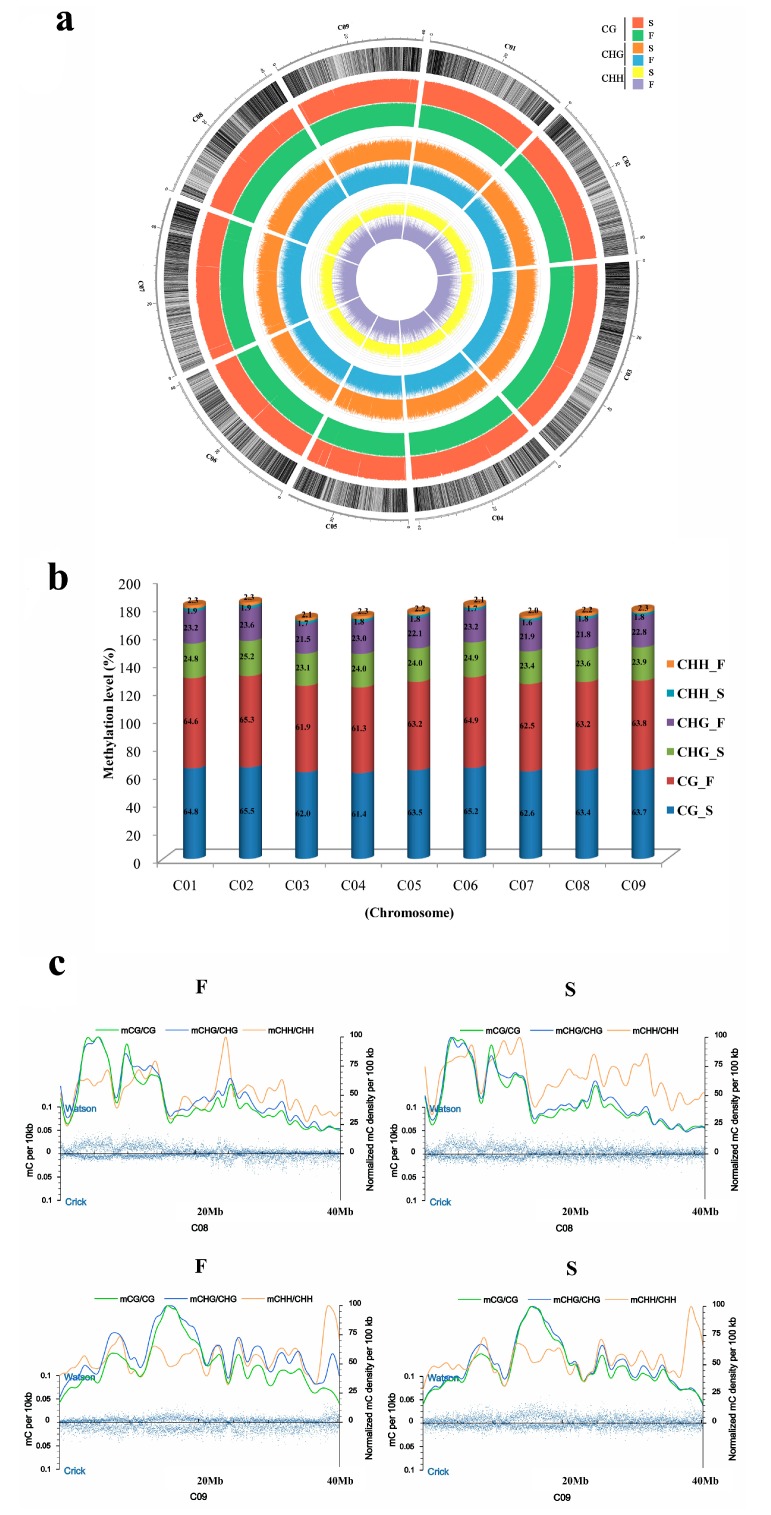
Genomic methylation levels of 01-20F and 01-20S. (**a**) Global DNA methylation patterns of 01-20F and 01-20S; (**b**) DNA methylation levels in nine chromosomes; (**c**) Methylcytosines density in chromosome 8 and 9. The blue dots represent methylcytosine density. The smoothed lines represent the methylcytosine density of the three contexts.

To show the genome wide DNA methylation landscape of the two samples, DNA methylation maps in the three contexts are represented using a Circos histogram (Figure 2a); some different methylation peaks were observed. We further calculated the percentage of DNA methylation levels in each context throughout the nine chromosomes (Figure 2b); these nine chromosomes showed similar methylation patterns to the whole genome. We speculated that there were some local changes in the DNA methylation levels. At a resolution of 10 kb, the methylation patterns are displayed using coordinating smoothed lines (Figure 2c), which confirmed these local changes. The methylation patterns of mCG, mCHG and mCHH in the F and S lines, e.g., some local positions of chromosomes 8 and 9, can be clearly distinguished.

Additionally, the methylation levels across various gene regions (e.g., upstream of the gene, the gene body (including the intron and exon), and the downstream region) were inspected. Relatively higher methylation levels were observed in the upstream and downstream regions than those observed in the gene body regions, similar to *Arabidopsis thaliana* [2,17] and another subspecies of *Brassica oleracea* named TO1000 [34]. However, unlike in TO1000, the levels in introns were not higher than those in exons. In this study, comparing the two libraries revealed that the S samples showed slightly decreased methylation levels in the upstream and downstream regions (Figure S1).

### 2.2. DNA Methylation Patterns of Genes

The differentially methylated genomic regions (DMRs) between the two lines were profiled. In total, 505 DMRs (*q* < 0.01) were detected. To understand the DNA methylation levels around genes, DMRs in the upstream 2 k, gene body (including introns and exons), and downstream 2 k regions were defined as DMR-associated genes. In total, 106 DMR-associated genes were identified, including 61 hypermethylated genes and 45 hypomethylated genes in the S background. Additionally, 43 genes were hypermethylated or hypomethylated in the upstream region, 32 in the gene body (including only three genes in exon regions), and 31 in upstream regions. These DMR-associated genes were annotated using BGI Web Gene Ontology Annotation Plotting, assigned to three major groups (i.e., biological process, cellular component, and molecular function), and annotated to be involved in cellular component and process, DNA binding, cation binding, carbohydrate metabolic process, organic substance transport, and other processes (Figure S2, Table S2).

### 2.3. The S Background Altered the Expression Levels of Some DMR-Associated Genes Involved in Pollen Development

Among the 106 DMR-associated genes, nine genes were found to have possible relationships with pollen development, and of these, eight were found to be hypermethylated and one to be hypomethylated in 01-20S. Three (*Bol037820*, *Bol016189*, and *Bol019810*) were hypermethylated at a location 2 k upstream, three (*Bol009558*, *Bol042253*, and *Bol007244*) were hypermethylated in introns, and two (*Bol037763*, *Bol039180*) were hypermethylated at a region 2 k downstream. One area (*Bol035575*) was hypomethylated 2 k downstream.

According to their annotations in the Brassica Database (BRAD) and the known homologous genes in *A. thaliana*, *Bol009558* (predicted endoglucanase), *Bol007244* (predicted endo-1, 4-beta-xylanase A), *Bol039180* (predicted invertase/pectin methylesterase inhibitor family protein (PMEI)), *Bol035575* (predicted pectinesterase, active site) may be involved in the assembly or degradation of cell walls, including the pollen wall and the PMC wall. *Bol037820* (*AT2G35210*, *E*-value = 1 × 10^−117^) is predicted to encode an adenosine diphosphate (ADP)-ribosylation factor (GTPase-active), which regulates pollen and pollen tube development. *Bol037763* (*AT2G34880*, *E*-value = 0.0, transcription factor jumonji) is a predicted maternally expressed imprinted gene involved in pollen development. *Bol042253* (*AT4G25640*, *E*-value = 0.0) is predicted to encode a multidrug and toxin efflux family transporter that is involved in flavonoid metabolism; is required for root growth, seed development, and germination; and plays a role in pollen development, release, and viability.

*Bol019810* (*AT1G34650*, *E*-value = 0.0, predicted homeobox-leucine zipper protein HDG10) and *Bol016189* (*AT1G70780*, *E*-value = 1 × 10^−55^, unknown protein) are expressed in the tapetum and pollen grains or pollen tube cell, but their functions remain poorly understood. These nine genes are listed in Table 1.

**Figure 3:**
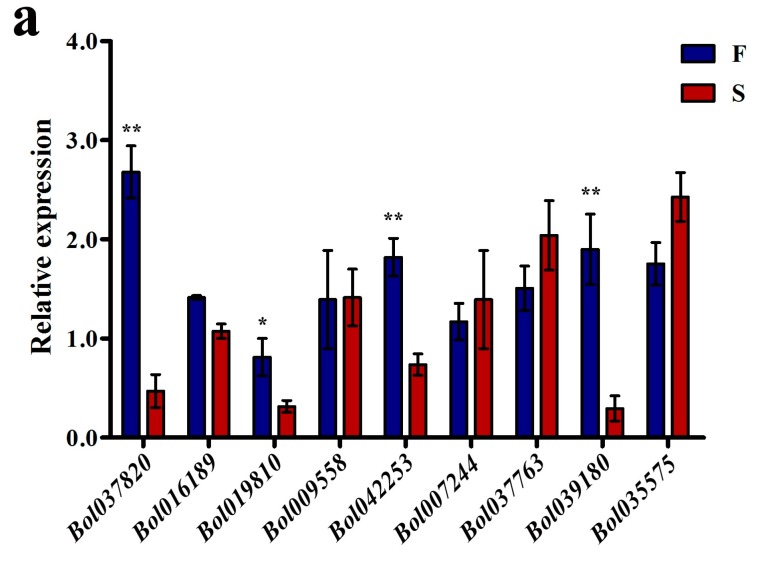

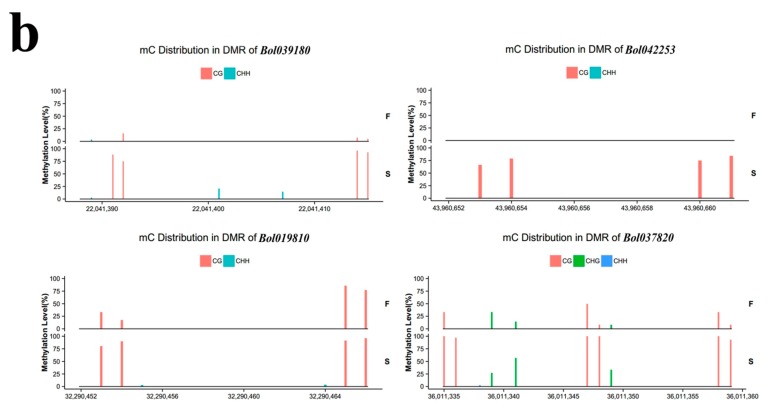
Gene expression and DNA methylation patterns of differentially methylated genomic regions (DMR)-associated genes involved in pollen development. (**a**) Expression analysis of nine genes related to pollen development; (**b**) DNA methylation at specific sites of four genes by bisulfite sequencing (BS-seq). The asterisk indicates a significant difference between F and S (* *p*-value < 0.05; ** *p*-value < 0.01).

Previous studies have suggested that high methylation levels across gene structures, especially in upstream regions, tend to be associated with lower gene expression levels [1,4,34]. To investigate the expression levels of the nine pollen-development associated genes in 01-20F and 01-20S, a quantitative reverse-transcription polymerase chain reaction (qRT-PCR) was performed. Two of the three upstream hypermethylated genes (*Bol037820* and *Bol019810*) were downregulated in 01-20S, while only one downstream hypermethylated gene (*Bol039180*) and one intron hypermethylated gene (*Bol042253*) were downregulated. The expression levels of the remaining five genes did not differ significantly. Methylation may play a role in regulating the expression of these genes. The methylation patterns and expression levels of the four differently expressed genes are shown in Figure 3. The primers used for qPCR are listed in Table S3.

### 2.4. Validation of BS-Seq by Bisulfite PCR

To validate the quality of the BS-Seq data, methylation-specific PCRs (bisulfite PCRs) were employed to identify the methylation levels of three DMR-associated genes (*Bol007715*, *Bol032135*, and *Bol019810*). In 01-20S, *Bol007715* was hypermethylated in intron, *Bol032135* was hypomethylated in the downstream region, and *Bol019810* was hypermethylated in the upstream region (Figure S3); these findings are in good agreement with the BS-Seq results.

## 3. Discussion

DNA methylation regulates many biological processes, including transposon silencing, genomic imprinting, DNA repair, and gene transcription [1,15]. Some studies have found altered methylation patterns in male sterile plants and evidence that methylation regulates EGMS in rice [23,24,25], CMS in rice [26] and maize [27] and GMS in tomato [28] and wheat [29]. However, to date, no study has reported these methylation profiles and their possible roles in the cabbage male sterility line.

In this study, the global DNA methylation pattern was profiled in the cabbage line 01-20F and its near-isogenic line (NIL) 01-20S using BS-Seq. This is the first global DNA methylation pattern to be determined in cabbages. This pattern was similar but slightly different to that of another morphotype in *B. oleracea*, a kale like plant named TO1000 (*B. oleracea* var. *oleracea*) [34]. Both genomes were enriched in mCG relative to *A. thaliana* (22%) [2,17]. In the cabbage genome, we observed higher levels of cytosine methylation for both mCG (~63%) and mCHG (22–24%) (Figure 1b) relative to TO1000 (54.9% for mCGs and 9.4% for mCHG) [34]. However, we demonstrated similar levels in mCHH (Figure 1b). Regarding the average cytosine methylation around genes, we also observed higher levels in the upstream and downstream regions in the cabbage, but did not find higher levels in introns than in exons (Figure S1), in contrast to TO1000 [34]. These differences may result from the different morphotypes and tissues used in these two studies.

When comparing 01-20F and 01-20S, the genomic cytosine methylation levels showed few differences, including a slightly lower methylation level at mCHH and a higher level at mCHG in 01-20S (Figure 1a,b). Some obvious local changes were also observed, indicating that the *Ms-cd1* background might alter the methylation pattern in some genomic fragments (Figure 2). DMRs between 01-20F and 01-20S were identified, with no biased number of hypermethylated (250) and hypomethylated (255) regions, in contrast to a report on the DNA methylation pattern of Cd-exposed rice [35]. The DMR-associated genes were analyzed. More DMR-associated genes were found to be hypermethylated than hypomethylated in upstream and downstream regions in 01-20S, which is very similar to the photoperiod- and thermo-sensitive male sterile rice described by Hu et al. [25].

The relationship between DNA methylation and gene structure/gene expression has been studied in many plants [1,4,34]. In this study, some genes affected by DNA methylation are differentially expressed in 01-20F and 01-20S. Some anther and pollen development-related genes with higher DNA methylation levels were downregulated in 01-20S, as detected by qRT-PCR. *Bol039180* is predicted to encode an invertase/PMEI that is likely involved in pectin degradation (BRAD database). Interestingly, Lou et al. [31] identified another PMEI in *Ms-cd1* background *B. oleracea* (broccoli and cabbage), and speculated that it plays a role in pollen separation defects on the *Ms-cd1* background of *B. oleracea.* The *MS-cd1* mutant gene may result in the suppressed expression of different PMEI genes, disrupting the separation of pollens from tetrads. *Bol037820* is homologous to *AGD10* (*AT2G35210*) in *A. thaliana* (BRAD database), encoding an ADP-ribosylation factor GTPase-activating protein. Several studies have reported that *AGD10* is only expressed in roots and pollens (pollen grains and pollen tubes) and is involved in the development of pollen, pollen tubes, and root hair [36,37], although its role is not well understood. *Bol019810* is homologous to *HDG10* (*AT1G34650*) in *A. thaliana* (BRAD database), and encodes a homeobox-leucine zipper protein that functions as a transcription factor. Nakamura et al. [38] found that *HDG10* is exclusively expressed in anthers, with higher expression levels in the tapetum and pollen grains, suggesting that it might play a role in pollen development. However, its T-DNA insertion mutant exhibited no abnormal phenotypes [38]. *Bol042253* (*AT4G25640*) is predicted to encodes a multidrug and toxin efflux family transporter *DTX35* (ABC transporter) (BRAD database), which is involved in flavonoid metabolism, root growth, seed development and germination, as well as pollen development, release, and viability [39,40]. Thompson et al. [40] reported that *AtDTX35* is required for pollen development, release, and viability; indeed, the absence of the *AtDTX35* mutant altered flavonoid metabolism by affecting the flavonoid levels, leading to shrunken and irregular pollen, aberrant pollen surface structure, and defects in anther dehiscence.

Moreover, some studies have reported that lipids [41,42], flavonoids [43,44], and the circadian rhythm [45] are involved in pollen development. Therefore, some other DMR-associated genes (e.g., *Bol038952* (putative lipid-transfer protein), *Bol042407* (predicted cyclopropane-fatty-acyl-phospholipid synthase), *Bol035147* (predicted consensus amino acid sequence of Gly, Asp, Ser, and Leu around the active site Ser (GDSL) esterase/lipase-like protein), *Bol039182* (predicted esterase/lipase/thioesterase family protein), *Bol038837* (predicted flavone 3’-O-MET), and *Bol021597* (predicted FAR1 family protein, positive regulation of circadian rhythm)) may also have potential roles in pollen development. These roles require further study.

## 4. Materials and Methods

### 4.1. Plant Materials

The 01-20F is an elite cabbage inbred, line bred though system selection from the variety ‘Early Vikings’ introduced from Canada by the Institute of Flowers and Vegetables of the Chinese Academy of Agriculture Sciences (IVFCAAS, Beijing, China).

The 01-20S, which harbors *Ms-cd1* introduced from 79-399-3, is a male sterile line generated through a cross between 01-20F and 79-399-3 and subsequent backcrossing with 01-20F for more than 20 generations. They were grown in a greenhouse at IVFCAAS, germinated under the condition of 25 °C, vernalized (after seven true leaves stages) under the condition of 1–4 °C for three months, and bolted and flowered under the condition of 25 °C. During the flowering stage, the flower buds were harvested from 01-20F and 01-20S separately. These samples were flash frozen in liquid nitrogen and stored in a −80 °C freezer.

### 4.2. Library Construction and BS-Seq

High quality genomic DNA was extracted from the buds using a QIAamp DNA Mini Kit (Qiagen, Valencia, CA, USA). The DNA quality was then evaluated by agarose gel electrophoresis and a spectrophotometer (BioDrop, UK). The two DNA samples were fragmented to approximately 200–300 bp by sonication using a Diagenome sonicator. The fragments were blunt-ended and phosphorylated. Subsequently, a single ‘A’ nucleotide was added to the 3’-end, and adaptor ligation was conducted according to the manufacturer’s instructions (Illumina, San Diego, CA, USA). The bisulfite conversion of the DNA fragments was performed using a ZYMO EZ DNA Methylation-Gold kit (NEB, Ipswich, MA, USA). The bisulfite-treated DNA fragments were purified on a 2% agarose gel. Finally, the two libraries were sequenced using the ultra-high throughput Illumina HiSeq 2500 platform.

### 4.3. Bioinformatic Analysis

The raw reads produced from BS-Seq were filtered to generate high-quality clean reads. DNA methylation is strand specific, resulting in the noncomplementarity of the two DNA strands. To build pre-converted forms of the reference genome, cytosine residues were converted to thymidine residues in the sense strand, whereas guanine residues were converted to adenosine residues in the antisense strand. The reference genome of ‘02-12’ cabbage is available at the *B. oleracea* Genomics Project [46]. The high-quality clean reads were then aligned to the prepared reference genome using Bismark [47]. Unique matches were filtered for further methylation analyses.

The individual methylated C sites were identified using an R package methylKit [48]. The DMRs between the two samples were screened according to the method of Li et al. [49]. To retrieve the DMRs, each certain (algorithm-specified) methylated genomic region needed to contain at least three C sites, with at least one differentially methylated C site (*Q*-value < 0.01). Those algorithm-specified regions with an absolute mean methylation difference greater than 10% between F and S were determined as DMRs. The two-sample *t*-test was performed to measure significant differences between F (01-20F) and S (01-20S) in terms of CG, CHG, CHH using SPSS 17.0 (IBM Corp., Armonk, NY, USA).

### 4.4. Bisulphite Sequencing PCR

To validate the BS-Seq results of the two samples, bisulfate PCR was performed. First, 1 μg of genomic DNA was treated with bisulfite using a CpGenome Turbo Bisulfite Modification Kit (Millipore Corp, Billerica, MA, USA). Primers for BS-Seq were designed using the Meth-Primer Program [50]. The PCR products were purified and cloned into pEASY-Blunt Zero Cloning Vector (TransGen Biotech, Beijing, China) for sequencing. In total, 20 clones were sequenced for each PCR product. The BS-Seq results were analyzed using the BiQ Analyzer software [51].

### 4.5. Real-Time RT-PCR

The total RNA was extracted from buds of 01-20F and 01-20S using an RNAprep pure Plant Kit (TIANGEN, Beijing, China) according to the manufacturer’s instructions. The RNA was then treated with RNase-free DNase I (Fermentas, Harrington, QC, Canada) to remove genomic DNA. The first-strand cDNA was synthesized using the PrimeScript 1st Strand cDNA Synthesis Kit (Takara, Kyoto, Japan).

The quantitative RT-PCR (qRT-PCR) reaction mixture was prepared using the SYBR R Premix Ex TaqTM II (Tli RNaseH Plus) kit (Takara), and the reaction was performed using a CFX96 Touch real-time PCR detection system (Bio-Rad, Hercules, CA, USA). The qPCR reactions were repeated in triplicate for three independent biological replicates. The PCR specificity was determined by melting curve analysis, and the relative expression was calculated using the 2^−ΔΔCT^ method [52]. A two-sample *t*-test was performed to assess whether the gene expression levels were significantly differential. The statistical analyses were performed with SPSS 17.0 (IBM Corp., Armonk, NY, USA).

## Figures and Tables

**Table 1:** Information on nine differentially methylated genomic region (DMR)-associated genes involved in pollen development.

ID	Different Methylation Region	Hyper or Hypo in S	*p*-Value	Homologous Gene in *A. thaliana*	*E* Value	Predicted Functional Description
*Bol009558*	Intron	hyper	1.74 × 10^−3^	*AT5G49720*	1.00 × 10^−16^	Endoglucanase 21
*Bol037820*	Upstream	hyper	5.09 × 10^−12^	*AT2G35210*	1.00 × 10^−117^	ADP-ribosylation factor GTPase-activating protein AGD10
*Bol016189*	Upstream	hyper	1.84 × 10^−7^	*AT1G70780*	1.00 × 10^−55^	Unknown protein; expressed in sperm cell, male gametophyte, pollen tube
*Bol042253*	Intron	hyper	2.76 × 10^−8^	*AT4G25640*	0.00	ABC transporter; multidrug and toxin efflux family transporter DTX35
*Bol007244*	Intron	hyper	1.98 × 10^−7^	*AT4G08160*	0.00	Endo-1,4-beta-xylanase A
*Bol019810*	Upstream	hyper	6.36 × 10^−5^	*AT1G34650*	0.00	Homeobox-leucine zipper protein HDG10
*Bol035575*	downstream	hypo	2.62 × 10^−5^	*AT1G53840*	0.00	Pectinesterase, active site
*Bol037763*	downstream	hyper	1.16 × 10^−5^	*AT2G34880*	0.00	Transcription factor jumonji
*Bol039180*	downstream	hyper	1.93 × 10^−11^	*AT2G47340*	0.38	invertase/pectin methylesterase inhibitor family protein

Hyper: Hypermethylated; Hypo: Hypomethylated; S: sterile line 01-20S.

## Supplementary Materials

The following are available online at www.mdpi.com/2073-4425/8/6/159/s1. Figure S1: Methylation profile in upstream of the gene, the gene body and the downstream region. Figure S2: GO analysis of differential methylated genes between 01-20F and 01-20S. Figure S3: Validation of the BS-Seq results by bisulfite methylation-specific PCR. Table S1: Output data of bisulfite sequencing (BS-Seq) of libraries F (01-20F) and S (01-20S). Table S2: Information of all the DMR-associated genes. Table S3: Primers for qPCR and BS-PCR used in this study.

## Abbreviations

BS-Seq	Single-base-resolution bisulfite sequencing
DMRs	Differentially methylated genomic regions
qRT-PCR	Quantitative reverse-transcription polymerase chain reaction
MET1	Methyltransferase 1
CMT3	Chromomethylase 3
DDM1	Decrease in DNA methylation 1
DME	DNA demethylases DEMETER
ROS1	Repressor of silencing 1
TEs	Transposable elements
PMC	Pollen mother cell
EGMS	Environmentally sensitive genic male sterile
LDMAR	Long-day-specific male-fertility-associated RNA
MSAP	Methylation-sensitive amplified polymorphism
MeDIP-seq	DNA immunoprecipitation sequencing
CMS	Cytoplasmic male sterility
DGMS	Dominant male sterility
BRAD	Brassica Database
PMEI	Pectin methylesterase inhibitor family protein
NIL	Near-isogenic line
GDSL	Consensus amino acid sequence of Gly, Asp, Ser, and Leu around the active site Ser
IVFCAAS	Flowers and Vegetables of the Chinese Academy of Agriculture Sciences
